# Structure and function of microbiomes in the rhizosphere and endosphere response to temperature and precipitation variation in Inner Mongolia steppes

**DOI:** 10.3389/fpls.2023.1297399

**Published:** 2023-12-07

**Authors:** Wenchen Song, Yao Wang, Bo Peng, Linyan Yang, Jian Gao, Chunwang Xiao

**Affiliations:** ^1^College of Life and Environmental Sciences, Minzu University of China, Beijing, China; ^2^Key Laboratory of Ecology and Environment in Minority Areas (Minzu University of China), National Ethnic Affairs Commission, Beijing, China; ^3^Faculty of Resources and Environment, Baotou Teachers’ College, Inner Mongolia University of Science and Technology, Baotou, China

**Keywords:** microbial communities, steppe ecosystems, rhizosphere and endophytic microorganisms, Inner Mongolian steppes, metagenomic sequencing

## Abstract

**Introduction:**

Owing to challenges in the study of complex rhizosphere and endophytic microbial communities, the composition and function of such microbial communities in steppe ecosystems remain elusive. Here, we studied the microbial communities of the rhizosphere and endophytic microbes of the dominant plant species across the Inner Mongolian steppes using metagenomic sequencing and investigated their relationships with changes in mean annual temperature (MAT) and mean annual precipitation (MAP).

**Methods:**

Metagenomic sequencing based on Illumina high-throughput sequencing, using the paired end method to construct a small fragment library for sequencing.

**Results:**

Adaptation of root systems to the environment affected the composition and function of rhizosphere and endophytic microbial communities. However, these communities exhibited distinct community assembly and environmental adaptation patterns. Both rhizosphere and endophytic microbial communities can be divided into two unrelated systems based on their ecological niches. The composition and function of the rhizosphere microbial communities were mainly influenced by MAT, while those of the endophytic microbial communities were mainly influenced by MAP. MAT affected the growth, reproduction, and lipid decomposition of rhizosphere microorganisms, whereas MAP affected reverse transcription and cell wall/membrane/envelope biogenic functions of endophytic microorganisms.

**Conclusion:**

Our findings reveal the composition and function of the rhizosphere and endophytic microbial communities in response to changes in MAP and MAT, which has important implications for future biogeography and climate change research.

## 1 Introduction

The root–microbe system is an important component of terrestrial ecosystems and one of the most important regulators of underground ecosystems (Miki, 2012). How do microbial communities and plant root systems jointly affect biogeochemical cycles in grassland ecosystems? What are their biological, spatial, and geographic patterns and driving factors? These questions have always been the focus of ecological and biogeography (Ochoa-Hueso et al., 2020; Zhang and Xi, 2021; Utami et al., 2022). Although some studies have revealed the patterns and principles of distribution and the factors driving underground microbial community structure (Chen et al., 2015; Vasar et al., 2022; Wu et al., 2023), research on the biogeographical patterns of endophytic and rhizosphere microbial communities remains rare. Endophytic and rhizosphere microbial communities are important links between plant and soil interactions (Attia et al., 2022; Utami et al., 2022). The rhizosphere, which lies within a few millimetres of the plant root system, is usually the most active site for soil microbes, often due to the organic matter that plants transport to the soil through their roots (Hassan et al., 2019; Kuzyakov and Razavi, 2019; Gregory et al., 2022). However, endophytic microbes live inside the plant root system, and, excluding a few parasitic microbes, they often have a symbiotic relationship with plants (Adeleke et al., 2023). Endophytic symbiotic microbes can play two roles: as “transporters” and “exchangers”. That is, they can transport various nutrients between the root system and soil, exchanging nitrogen, phosphorus, and potassium from the soil with the photosynthetic products provided by plants (Song et al., 2020; Adeleke and Babalola, 2021; Adeleke and Babalola, 2022). Therefore, studying the functional connections between endophytic and rhizosphere microbial communities in steppe ecosystems is of great significance for microbial ecology, grassland science, and biogeography.

The pH, water, oxygen, nutrient content, composition and concentration of microbial growth substrates, in addition to presence of antibacterial compounds and plant hormones within the rhizosphere, all shape the environmental conditions for rhizosphere microbes. Importantly, such conditions are markedly different from those of non-rhizosphere soil (Hinsinger et al., 2009; Xiong et al., 2021). Compared to the microbial community in non-rhizosphere soil, rhizosphere communities exhibit higher density, larger cell size (Deangelis et al., 2009; Lopes et al., 2016), greater microbial activity, and a higher turnover rate (Dennis et al., 2010; Reinhold-Hurek et al., 2015; Liu et al., 2022). Therefore, plant-microbial productivity in the rhizosphere markedly contributes to the global nutritional cycle (Ma et al., 2023). The rhizosphere microbial community considerably affects plant growth, induces systemic resistance, directly suppresses plant pathogens (Chowdhury et al., 2021; Pande et al., 2021), and affects nutrient cycling in the soil (Hacquard et al., 2022). Endophytic microorganisms live inside the roots. These organisms often invade plant roots and help plants obtain nutrition and exchange nutrients in the soil (Mushtaq et al., 2023). For example, arbuscular mycorrhizal fungi directly extend their hyphae into the root cells, which can in turn increase the nutrient absorption capacity of plants several fold (Staddon et al., 2020). Although some field studies have attempted to explain the adaptability of root and microbial systems to the environment, findings have often differed significantly. For example, grassland plants and soil microbial biomass are positively correlated with water content (Chen et al., 2015), while another study suggested that water content does not affect plant and soil microbial biomass (Zhang and Xi, 2021). In addition, a previous report posited that the effect of drought on plant and soil microbial biomass depends on soil texture (Vieira et al., 2019). A careful analysis of the above-mentioned studies revealed that the main reason for such discrepancies is that they did not consider the relationship between endophytic and rhizosphere microbial communities, resulting in incomplete evidence chains, which led to a one-sided understanding and misinterpretation of the results (Yu and Hochholdinger, 2018; Vasar et al., 2022). Focusing on the compositional and functional changes between endophytic and rhizosphere microbial communities in steppe ecosystems is therefore necessary.

The vast steppes of Inner Mongolia are an important livestock production base in China as well as a crucial ecological barrier in the North and Northeast regions (Zhang et al., 2020). In the context of global climate change, the root-microbe interaction driven by temperature and moisture changes is bound to respond. Thus, maintaining the ecological functions of Inner Mongolian grasslands would represent a significant challenge. In this case, the root and microbial systems of essential steppe ecosystem components should receive further attention. However, discrepancies in the regularity of root microbial communities in response to changes in MAT and MAP remain between studies. Some studies suggest that, as temperature and moisture increase, so does microbial activity, which leads to a corresponding increase in the respiration rate of root microorganisms (Yao et al., 2017; Wang et al., 2021; Yu et al., 2021). Other studies suggest that the root microbial community is driven by the roots, and increased temperature promotes photosynthesis, which in turn increases plant carbon input underground, promoting the activity of root microbial communities (Ma et al., 2014; Song and Zhou, 2021; Li et al., 2023). In this case, plants usually prioritize the supply of major underground carbon sources to symbiotic microorganisms such as mycorrhizal fungi, promoting the accumulation of their cell walls and other refractory organic matter, thereby promoting soil carbon sequestration (Liang, 2020; Zheng and Song, 2022; Li et al., 2023). In addition, when the water content increases, there are usually more available nutrients in the soil, which reduces the supply of nutrients to rhizosphere microorganisms and inhibits their activity (Ma et al., 2014; Song et al., 2018). Thus, determining which situation is more realistic in the root-microbe system of the Inner Mongolia steppes represents an important issue worth studying.

Previous studies have mainly focused on the compositional changes in soil microorganisms, with little research on the functional changes in endophytic and rhizosphere microbial communities (Xun et al., 2021). Metagenomic analysis can not only assess compositional changes in microbial communities, but can also infer functional changes in microbial communities (Vieira et al., 2021). In the present study, we employed metagenomic analysis to conduct a regional-scale pattern and driving factor study of the endophytic and rhizosphere microbial communities in 15 grassland samples in Inner Mongolia. These steppes cover a wide range of community types and abiotic conditions (climates and grasslands). Due to the continuous environment in Inner Mongolia, transect studies can reflect changes in microbial community composition and function with temperature and moisture (Chen et al., 2015). The aim of the present study was to determine how the composition and function of endophytic and rhizosphere microbial communities respond to MAT and MAP. If this response is dominated by the roots, then the structure and function of the root microbial community would mainly be influenced by the changes in symbiotic microbes with MAT and MAP. Conversely, root microbial community structure and function would be consistent with soil microbial community structure and function as MAT and MAP change. Our study provides first-hand data on the composition and function of root and rhizosphere microbial communities in the Inner Mongolian steppe ecosystems, which could facilitate the conservation and management of the ecosystems, particularly in the wake of climate change.

## 2 Methods

### 2.1 Experimental sampling

**Figure 1:**
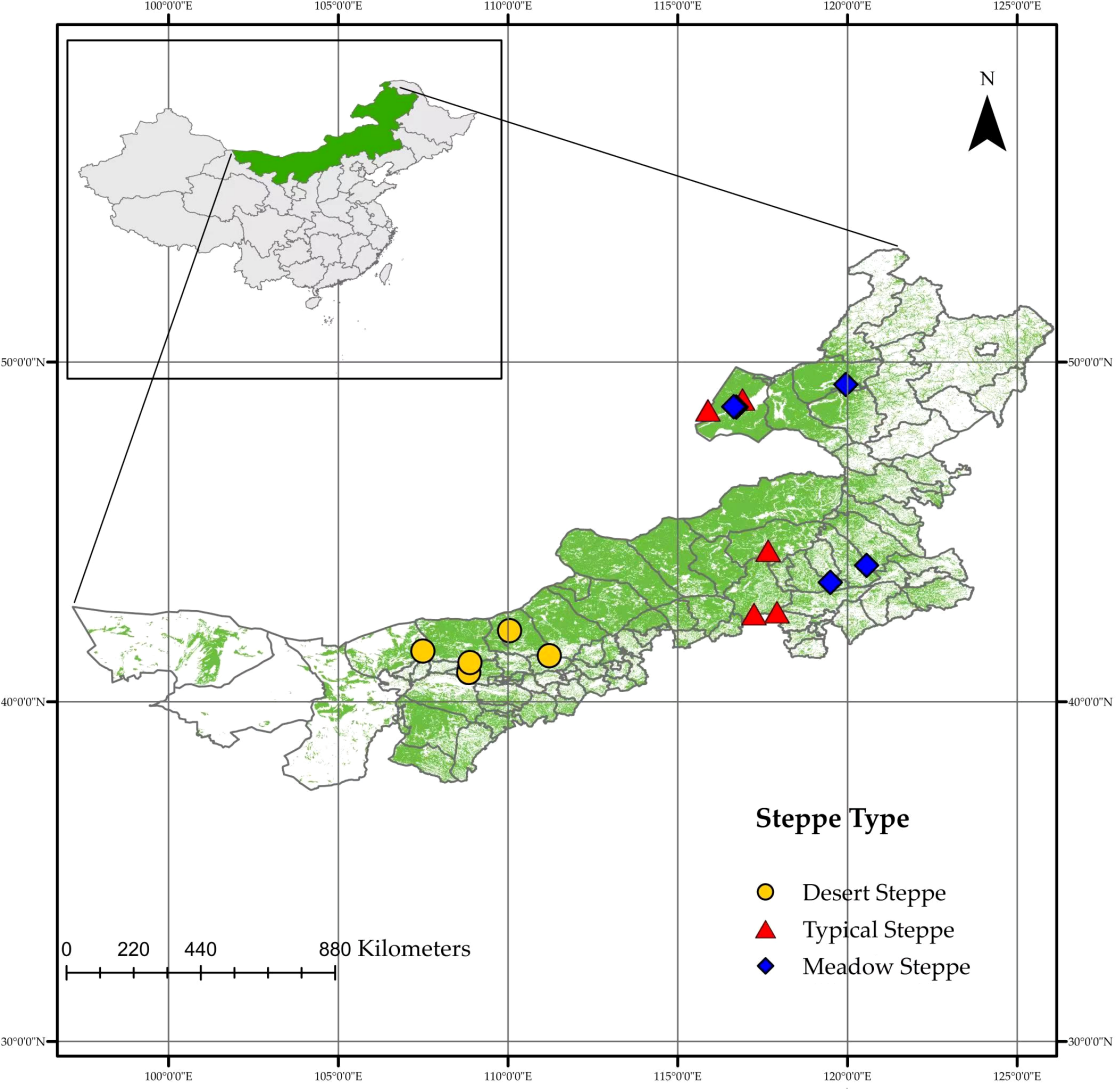
Map showing sampling sites within 15 steppes in Inner Mongolia.

Fifteen steppes located across a range of latitudes (40.86°–48.91°N) and longitudes (107.49°–120.56°E) in Inner Mongolia were surveyed during the summer of 2022 (**Figure 1**, **Table S1**). An approximately1,800-km long regional-scale transect was established across the MAT and MAP gradients on the Mongolian Plateau covering three major vegetation types: meadow steppe, typical steppe, and desert steppe. The steppes are typical representatives of the natural climatic top community which having typicality of climate and vegetation and can be used as stations for the transect (Chen et al., 2015). In each reserve, 40–50 plants of similar size from the dominant species (*Stipa* spp. and *Leymus chinensis*), which accounted for 80–100% of plants in each steppe, were picked randomly for root collection. We first randomly selected a plant and then randomly selected another one of similar size 3 m away, taking samples from both until we filled three 50-mL sampling bottles. Each plant chosen had one to three roots of 5-mm thickness from a 0–10-cm layer of soil. The samples were mixed into three composite samples for each sampled steppe. These roots were collected and stored in a Drikold bucket at −78°C. The data on MAT and MAP were downloaded from the National Meteorological Science Data Centre of China (http://data.cma.cn).

### 2.2 Separation of rhizosphere soil and pure root

Root samples were loaded into 2-mL sterile tubes filled with sterile water. The roots were shaken for 30 min and then removed from the tubes. The rhizosphere soil that adhered to the roots was separated after 25 min via centrifuging in sterile tubes at 15000 r min^-1^. The roots were then removed from the tube and washed three times with sterile deionised water to clean the root surface. The rhizosphere soils and pure root samples obtained were stored at −80°C for subsequent analysis.

### 2.3 Microbial DNA extraction and metagenome sequencing

Genomic DNA was extracted using a PowerSoil DNA Isolation Kit (Mo Bio Laboratories, Carlsbad, CA, USA). A Qubit (Invitrogen, Carlsbad, CA, USA, v.3.0, Qubit dsDNA HS Assay Kit) was used to detect the concentration of the extracted nucleic acids and agarose gel electrophoresis (1% agarose gel) was used for integrity detection. The qualified indicators employed were as follows: concentration ≥ 1 ng µL^-1^, total amount ≥ 0.1 µg, integrity mainly dispersed bands above 5 kb, and no evident impurities below. Metagenomic sequencing libraries were generated using the TrueLib DNA Library Rapid Prep Kit for Illumina (Vazyme Biotech Co., Ltd., China), according to the manufacturer’s recommendations. Sequencing was performed using a NovaSeq 6000 system (Illumina, San Diego, CA, USA). The library was fragmented using Qsep-400, and the library concentration was quantified using Qubit 3.0. The indicators for on-machine detection were: concentration ≥ 1 ng µL^-1^, quality inspection fragment center value between 430–530 bp, an average value between 420–580 bp, a peak-shaped normal distribution, and a single fragment without impurities.

### 2.4 Metagenome sequencing analysis

Trimmomatic software (v0.33, PE LEADING:3 TRAILING:3 SLIDING WINDOW:50:20 MINLEN:120) was used to filter the Raw Tags and obtain Clean Tags. Bowtie2 (v2.2.4, seed 123456 -I 200 -X 1000 –un-conc) was used to align Clean Tags to the host genome and remove host contamination. MEGAHIT (v1.1.2, default parameters) was used for metagenomic assembly (Li et al., 2015), and contig sequences shorter than 300 bp were filtered out. The assembly results were evaluated using QUAST (v2.3, with default parameters) (Gurevich et al., 2013). MetaGeneMark (v.3.26, default parameters A-D-f G) was used to identify coding regions in the genome (Tang et al., 2013). MMseq2 (Version 11-e1a1c) was used to remove redundancy with a similarity threshold of 95% and a coverage threshold of 90%.

Functional genes were classified and annotated using the eggNOG database, which is especially beneficial for analyzing functional diversity and the correlations between functional genes (Huerta-Cepas et al., 2019). The protein sequences of non-redundant genes were compared to the eggNOG database using BLAST (diamond v0.9.29, E-value 1e-5). The annotation and classification information of the most similar sequences in the database were then used for the corresponding sequenced gene.

### 2.5 Statistical analysis

The distribution map of the steppes was drawn using ArcGIS 10.2 (ESRI Inc., CA, USA). Python (v3.9) was used to draw species bar charts at the kingdom and order levels. The R (v4.0.2) vegan package was used to calculate the microbial Shannon index, a parameter suitable for large-scale ecological research (Song, 2023). The R package metagenomeSeq was used to perform the differential analysis of functional gene abundance and to draw an abundance heatmap (Paulson et al., 2013). The R packages “psych” (function: pearson) and “pheatmap”(function: average) were used to analyze the correlation between functional gene abundance and environmental factors as well as to draw a heatmap. The Spearman algorithm was used to perform correlation analysis and statistical testing based on the abundance and variation of each functional gene or taxon in each sample. The data groups with correlation > 0.5 and p-value < 0.05. A correlation network diagram was drawn using Python.

## 3 Results

### 3.1 Biogeographic variations

**Figure 2:**
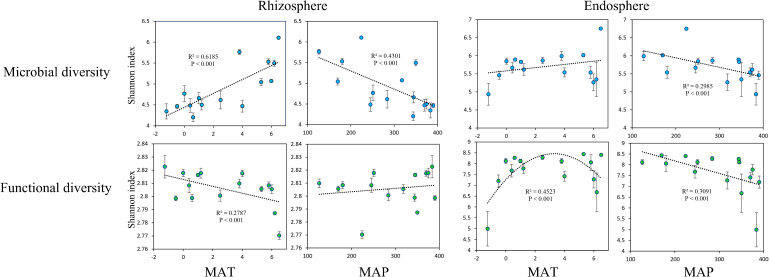
Relationships of mean annual temperature (MAT; °C) with microbial Shannon index and functional Shannon index, as well as mean annual precipitation (MAP; mm) with microbial Shannon index and functional Shannon index, in the rhizosphere and endosphere.

The microbial Shannon index in the rhizosphere was significantly positively correlated with MAT (**Figure 2A**). Meanwhile, the functional Shannon index in the rhizosphere exhibited a significant negative correlation with MAT (**Figure 2B**). The microbial Shannon index in the rhizosphere decreased significantly as the MAP increased (**Figure 2C**), and no significant relationship was found between the functional Shannon index in the rhizosphere and MAP (**Figure 2D**) nor between the microbial Shannon index in the endosphere and MAT (**Figure 2E**). The functional Shannon index of the endosphere was significantly and unimodally related to MAT (**Figure 2F**). However, the microbial and functional Shannon indices in the endosphere decreased significantly as the MAP increased (**Figures 2G, H**).

**Figure 3:**
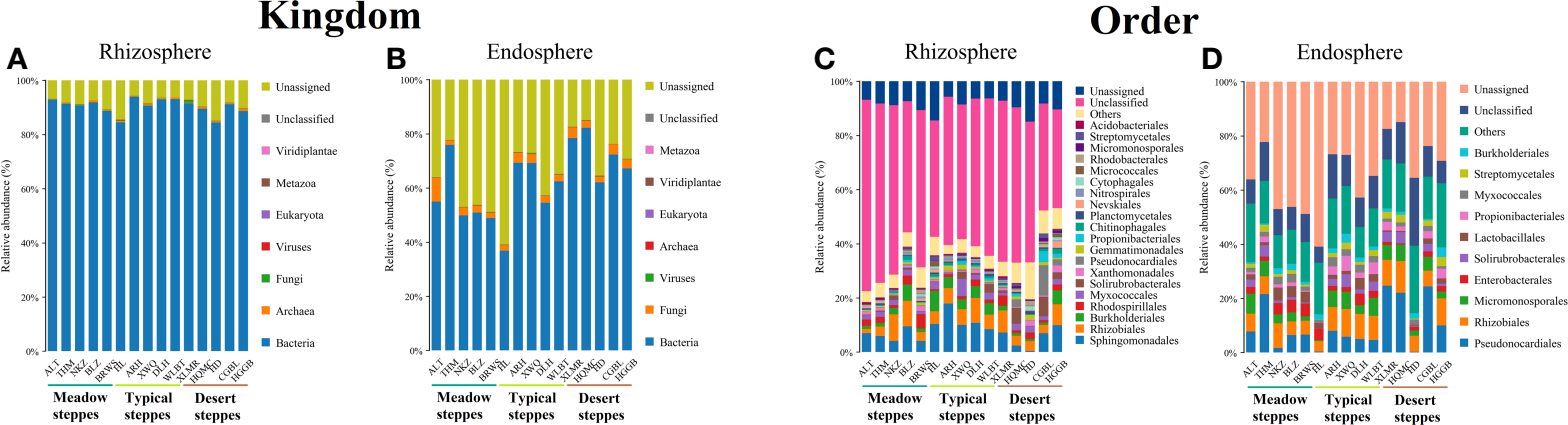
Distribution of kingdom-level microbial communities in **(A)** rhizosphere and **(B)** endosphere. Distribution of order-level microbial communities in **(C)** rhizosphere and **(D)** endosphere.

**Figure 4:**
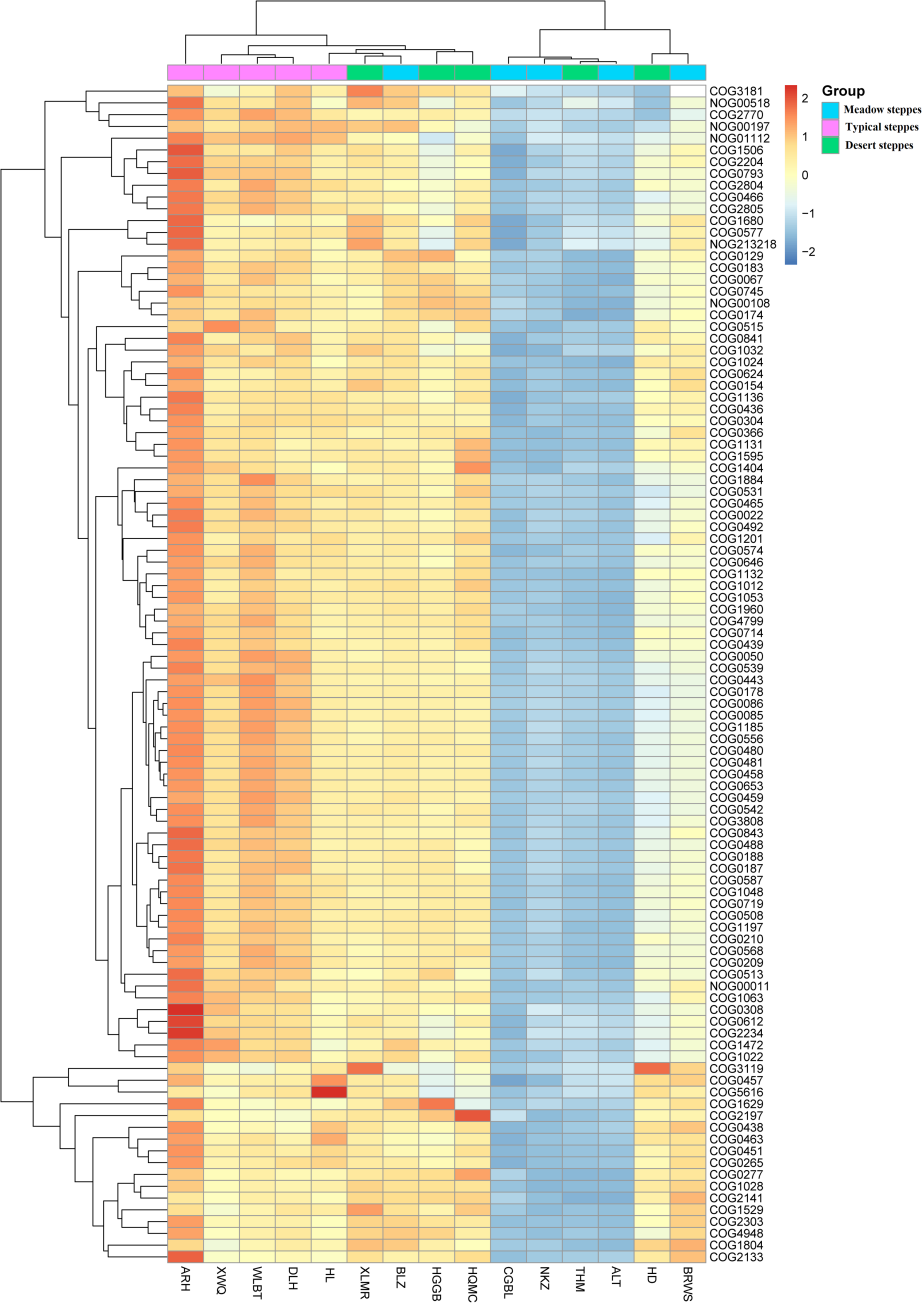
Heatmap of metagenomeSeq abundance of functional genes (eggNOG, top100) across microbial communities in the rhizosphere. Meaning of the functional gene database ID, description, class, and category shown in **Table S2**.

**Figure 5:**
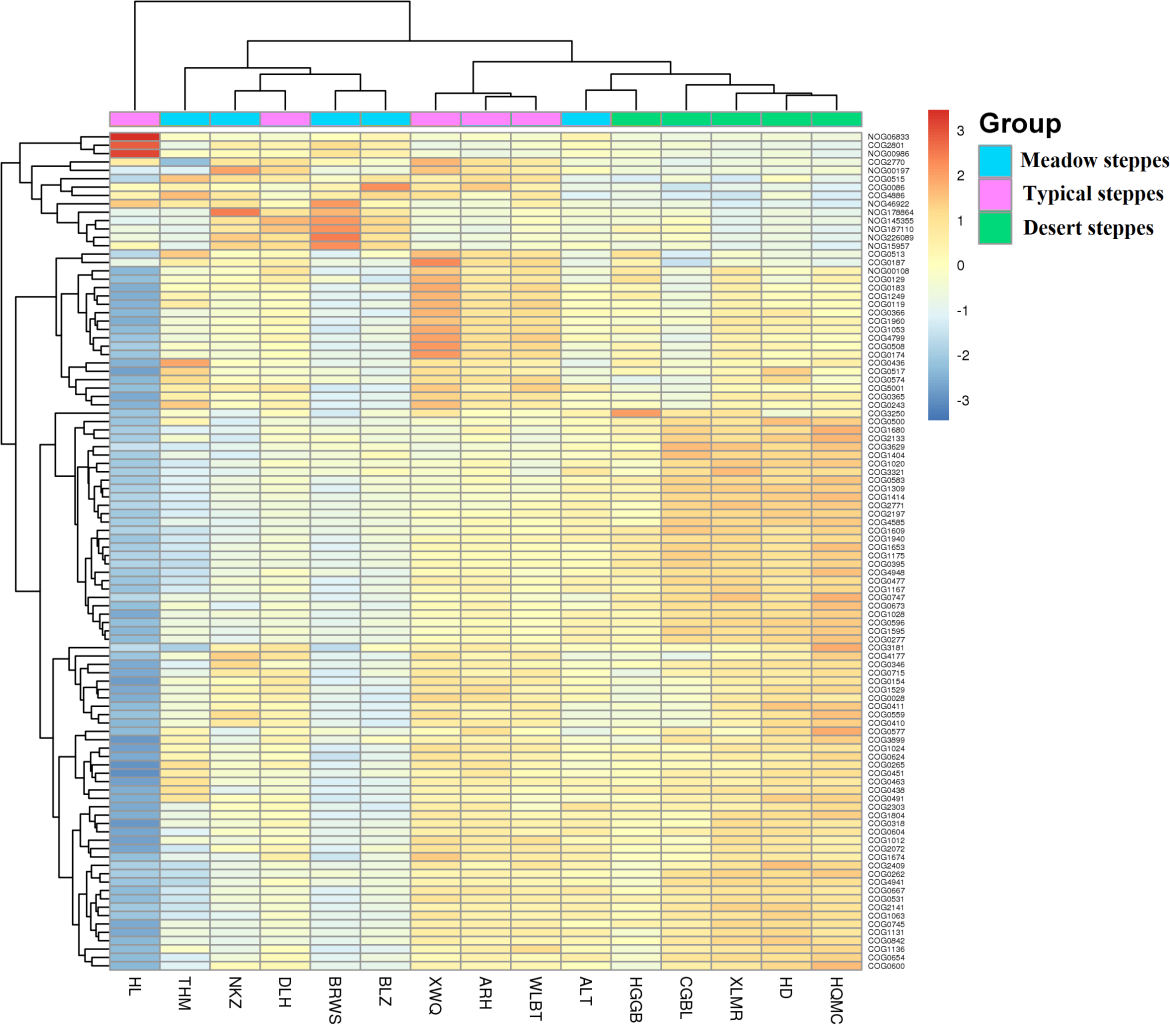
Heatmap of metagenomeSeq abundance of functional genes (eggNOG, top100) across microbial communities in the endosphere. Meaning of the functional gene database ID, description, class, and category shown in **Table S2**.

Bacteria dominated the microbial community in both the rhizosphere and endosphere; however, fungi were significantly more abundant in the endosphere than in the rhizosphere (**Figures 3A, B**). *Rhizobiales* and *Sphingomonadales* were the dominant orders in the rhizosphere (**Figure 3C**), whereas *Rhizobiales*, *Pseudonocardiales*, and *Micromonosporales* were the dominant orders in the endosphere (**Figure 3D**). When compared, the relative abundance of *Rhizobiales* in the different steppe types was not significantly different (**Figures 3C, D**). Changes in functional gene abundance in the rhizosphere and endosphere of different steppes are shown in **Figures 4** and **5**, respectively. Functional gene abundance in the rhizosphere was similar in typical steppes but not necessarily in other types (**Figure 4**). Similarly, functional gene composition in the endosphere was similar in desert steppes but not necessarily in other types (**Figure 5**).

### 3.2 Relationships of microbial functions

**Figure 6:**
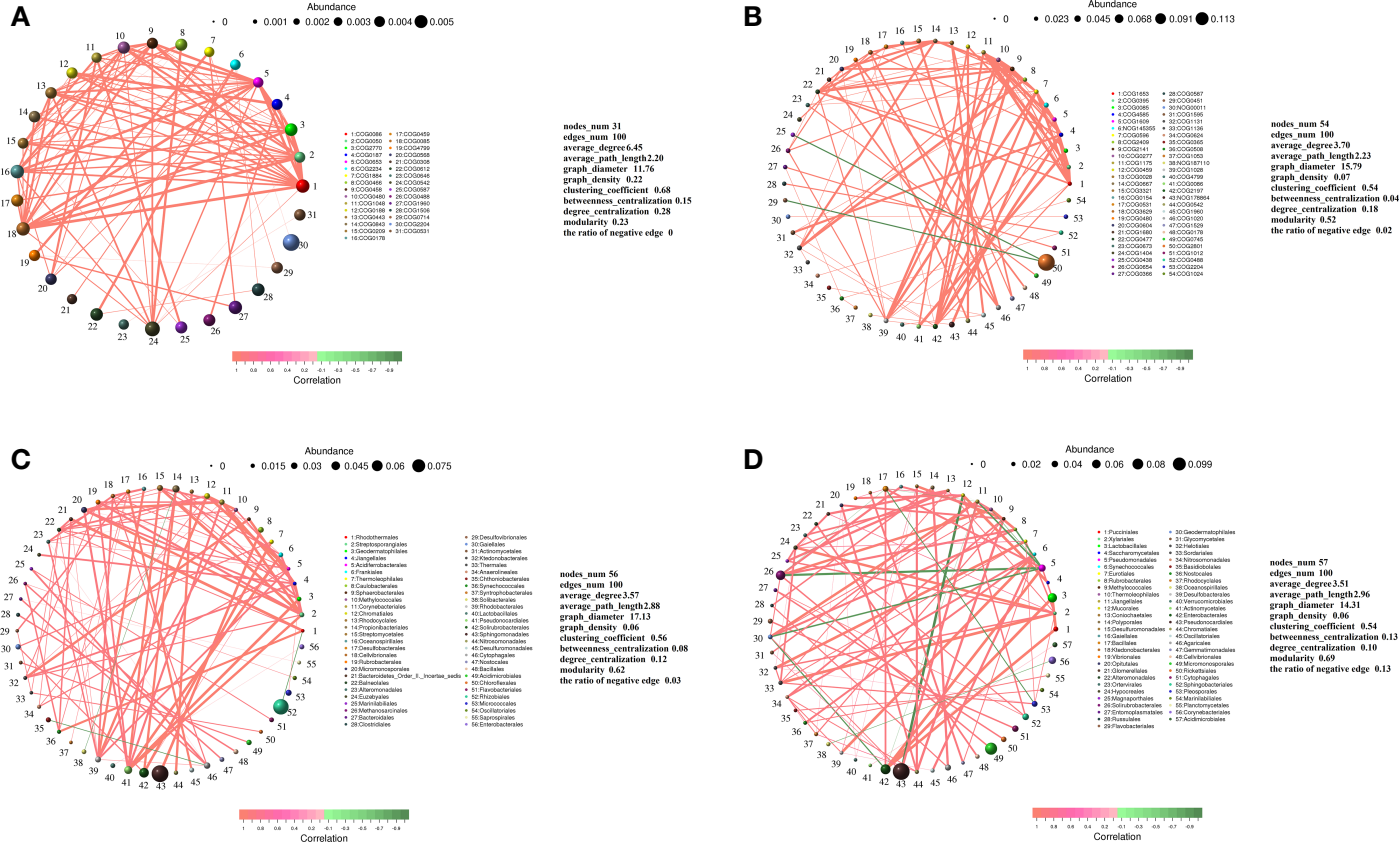
Correlation network diagram of functional genes in the **(A)** rhizosphere and **(B)** endosphere. Correlation network diagram of order-level microbial taxa in the **(C)** rhizosphere and **(D)** endosphere. Meaning of the functional gene database ID, description, class, and category shown in **Table S2**.

The relative abundances of different functional genes of rhizo-microorganisms varied slightly, in addition to exhibiting positive correlations among them (**Figure 6A**). In the endosphere, only the abundance of COG2801 (Retrotransposon protein) was much higher than that of other functional genes, and it exhibited a weak negative correlation only with COG0438 (Glycosyltransferase Group 1) and COG0451 (NAD-dependent epimerase dehydratase) (**Figure 6B**). Nevertheless, regardless of whether it was in the rhizosphere or endosphere, the highest relative abundance of the classified taxa exhibited a few significant correlations with other taxa. For instance, taking the order level as an example, in the rhizosphere, the highest abundance of *Rhizobiales* had a very weak negative correlation with *Rhodothermales*, and the second-highest abundance of *Sphingomonadales* had a positive correlation with *Caulobacterales* (**Figure 6C**). Similarly, in the endosphere, the second-highest abundance of *Pseudonocardiales* had a positive correlation with *Jiangellales* and a negative correlation with *Mucorales* (**Figure 6D**). *Micromonosporales*, with the third highest abundance, only had a positive correlation with *Solirubrobacterales* (**Figure 6D**), and *Rhizobiales*, with the highest abundance, was not correlated with other taxa (hence, it does not appear in the figure).

**Figure 7:**
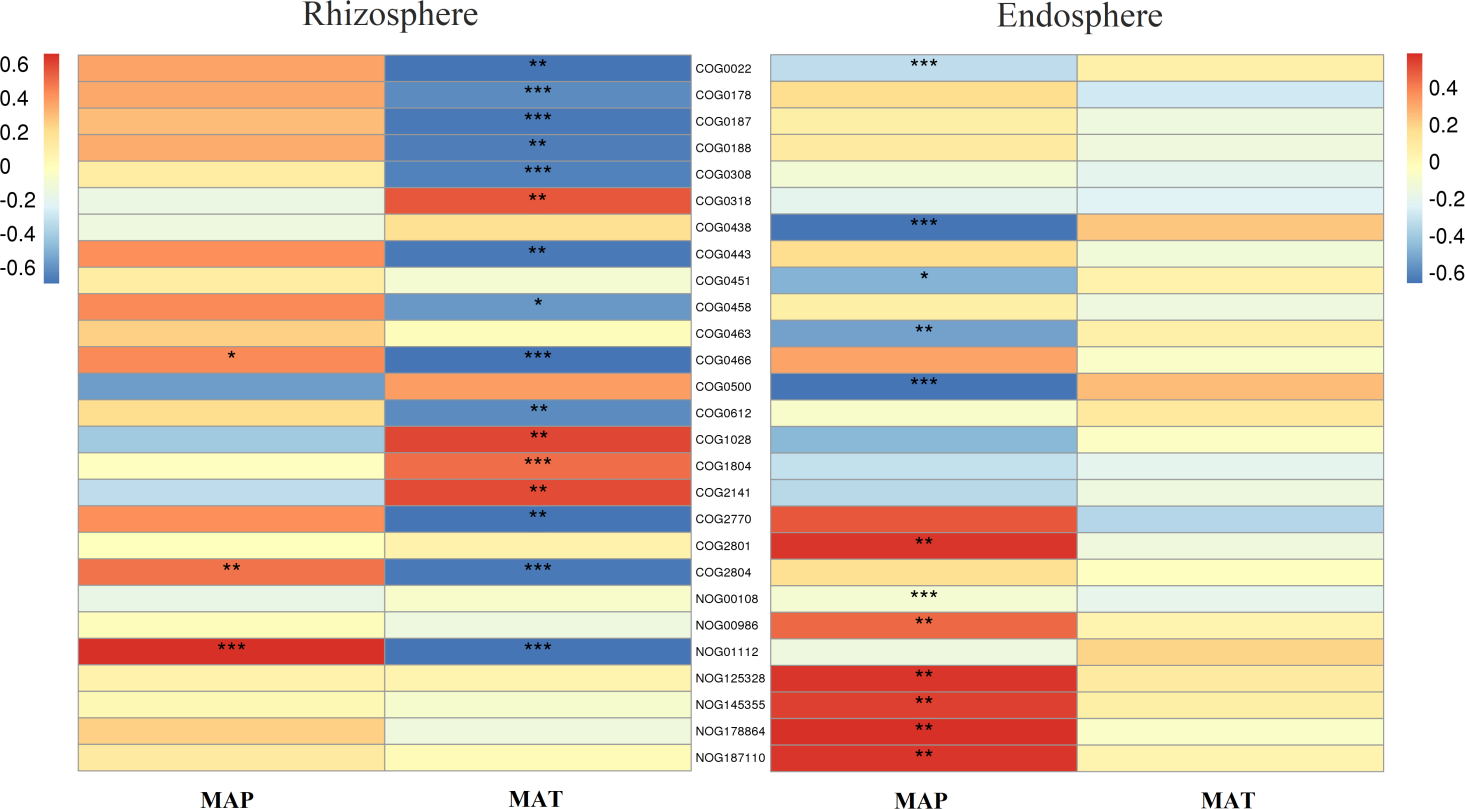
Correlation heatmap of main functional genes with mean annual temperature (MAT; °C) and mean annual precipitation (MAP; mm) in the rhizosphere and endosphere. Non-correlated functional genes are not shown. Significance: * <0.05; ** <0.01; *** <0.001. Meaning of the functional gene database ID, description, class, and category shown in **Table S2**.

Furthermore, in the rhizosphere, functional genes related to the modification, turnover, metabolism, and transport of microbial cell proteins (such as COG0308, COG0443, COG0466, COG0612, COG2770, and COG2804), partially related to microbial energy production and conversion (such as COG0022 and NOG01112), as well as genes related to DNA synthesis and transport (such as COG0022, COG0458, COG0187, and COG0188) were significantly negatively correlated with MAT (**Figure 7**). Meanwhile, those related to lipid metabolism (such as COG0318, COG1028, COG1804, and COG2141) exhibited a significant positive correlation with MAT (**Figure 7**). Notably, even though a few functional genes (such as COG0466, COG2804, and NOG01112) were significantly negatively correlated with MAP, in contrast to their relationship with MAT, most functional genes had no significant relationship with MAP (**Figure 7**).

In the endosphere, functional genes related to microbial cell wall/membrane/envelope biogenesis (such as COG0438, COG0451, COG0463, COG0500, and COG0843) and partially related to microbial energy production and conversion (such as COG0022 and NOG00108) were significantly negatively correlated with MAP (**Figure 7**), while those related to reverse transcription (such as COG0318, NOG00986, NOG125328, NOG145355, NOG178864, and NOG187110) were significantly positively correlated with MAP (**Figure 7**). Almost all functional genes showed no significant relationship with MAT (**Figure 7**).

## 4 Discussion

### 4.1 Variations in microbial communities

Both the rhizosphere and endophytic microbial communities are strongly influenced by plant roots, and changes in their composition are closely related to the plant–microbe interactions in response to climate change (Philippot et al., 2013; Song et al., 2022; Adeleke et al., 2023). As with most ecosystems, the observed rhizosphere microbial diversity was significantly and positively correlated with MAT. As temperature increases, plants transport more organic matter underground through their roots, increasing microbial activity, which manifests as a positive correlation between rhizosphere microbial diversity and temperature (Pausch and Kuzyakov, 2018; Dong et al., 2021; Zheng and Song, 2022). However, no significant relationship was observed between the Shannon index of the endophytic microbial communities and MAT, indicating that temperature was not the main factor influencing the communities. In contrast to the situation in non-rhizosphere soils, both rhizosphere and endophytic microbial diversity were significantly negatively correlated with MAP. We attributed the finding to the fact that high soil moisture content often means more available mineral nutrients are dissolved and released, which reduces plant dependence on nutrient uptake by microbes, thereby reducing the transport of organic matter underground and decreasing microbial diversity associated with roots (Song et al., 2018).

Regardless of whether in the rhizosphere or endosphere, relationships between microbial taxa showed similar characteristics, with dominant taxa abundance being absolute and having little correlation with the other groups. For example, *Rhizobiales* had a very weak negative correlation with *Rhodothermales* in the rhizosphere and no correlation with other groups in the endosphere. Therefore, as most *Rhizobiales* bacteria are closely associated with plant roots, and some even form symbiotic relationships with them (Garrido-Oter et al., 2018), they may only need to be closely associated with plants to gain a competitive advantage without establishing relationships with other microbial groups.

Non-dominant taxa compete for the remaining ecological niches of the dominant taxa. In other words, both the rhizosphere and endophytic microbial communities can be divided into two unrelated systems: one system is closely associated with the root system where the dominant species are located, and the remaining ecological niche system is closely associated with other microbes. Therefore, the abundance and composition of the dominant taxa, such as Rhizobiales, could represent steppe biomarkers.

The results of this study indicate that response patterns in the diversity and structure of rhizosphere and endophytic microbial communities to changes in MAT and MAP are different from those of soil microbial communities. That is, the diversity and structure of rhizosphere and endophytic microbial communities are more regulated by plants as a function of MAT and MAP, which means that plant adaptation to changes in temperature and precipitation dominates alterations in diversity and structure. Therefore, when studying and addressing the impact of global changes on the rhizosphere-microbial system, the role of a few dominant microorganisms, such as Rhizobiales, should be mainly considered, excluding interference from most other microorganisms.

### 4.2 Variations in microbial function

In the endosphere, only one type of functional gene was dominant and was rarely associated with other functional genes. This finding indicated that the functional genes of root-associated microbiota were mainly related to the root system. Surprisingly, retrotransposon genes were the dominant functional genes in the endosphere. The retrotransposons of root-associated microbiota can promote the expression of genes for adaptation to the plant root system environment, thereby helping plants resist pathogens and maintain endophytic microorganism diversity (Bavol et al., 2014; Vangelisti et al., 2019; Yadav et al., 2019). From a molecular ecology perspective, since most endophytic microorganisms rely on the root system for nutrient supply, retrotransposon function, which can be coordinated with plant regulation, emerges as the most important ecological adaptation function, with functional genes related to the retrotransposon process becoming dominant. However, the functional genes of rhizo-microorganisms were positively correlated, indicating that the overall trends of microbial functional change in the rhizosphere were consistent, as rhizo-microorganisms are not “picky” with regard to nutrient sources and can also utilize soil organic matter if the root supply is lacking, which confers them with greater adaptability (de la Fuente Cantó et al., 2020; Fan et al., 2022; Liu et al., 2022), resulting in different functional genes with no significant differences in abundance and consistent trends.

In the rhizosphere, functional genes related to the modification, turnover, metabolism, and transport of microbial cells as well as functional genes related to DNA synthesis and transport were significantly negatively correlated with MAT. The finding indicated that rhizosphere microorganisms in colder regions tend to use nutrients for growth and reproduction. However, owing to the lower organic matter content and more difficult utilization of soil in colder regions, the rhizosphere microbial community tends to reinforce its connection with plants, thereby increasing the functional diversity of the rhizosphere microbial community and enhancing its ability to utilize rhizosphere nutrients (Wyatt et al., 2014; Liu et al., 2020; Song and Zhou, 2021). This finding also explains why rhizo-microbial diversity was significantly and positively correlated with MAT and why the functional diversity of rhizosphere microorganisms was significantly and negatively correlated with MAT. Conversely, in warmer regions, rhizosphere microorganisms are relatively more abundant and active (Lv et al., 2023). Therefore, functional genes related to decomposition metabolism, especially lipid decomposition, were significantly positively correlated with MAT.

The functional diversity of the rhizosphere microbial community was not significantly related to the MAP, indicating that water was not the main factor affecting microbial function. This finding could be attributed to the fact that rhizo-microorganisms do not necessarily depend on roots and can make certain adaptive adjustments (Bhattacharyya et al., 2021; Angulo et al., 2022), meaning that the regulatory role of the rhizo-microbial community on water factors is relatively flexible. Similar principles can also explain why the functional gene composition of rhizo-microorganisms is similar in typical steppes, mainly due to the water and thermal conditions of typical steppes being relatively suitable, and the rhizo-microbial community has less need for adaptive adjustments tailored to local conditions.

Unlike the function of the rhizo-microbial community, endophytic microbial community function is driven by roots (Adeleke et al., 2023). Owing to the ecological amplitude variation in plant adaptation to temperature, the diversity of endophytic microbial function also showed significant unimodal variation with MAT (Muller-Landau et al., 2021). As plants rely on root-associated microbes to a greater extent in drier areas, the diversity of endophytic microbial functions is significantly negatively correlated with MAP (Soussi et al., 2016), and the endophytic microbial functional composition of desert steppes is similar. In addition, functional genes related to the cell wall, membrane, and envelope biogenesis in endophytic microbes were also significantly negatively correlated with MAP. An increase in these functional genes in the root circle usually indicates an increase in difficult-to-decompose substances, such as hyphae, cell walls, and cell envelopes generated by endophytic microbes (especially fungi and *Rhizobiales*), most of which are eventually transformed into microbial necromass and sequestered (Joergensen and Wichern, 2008; Joergensen, 2018; Wang et al., 2023). Therefore, a decrease in precipitation may enhance the “entombing effect” of microbes and the carbon sequestration capacity of the root system (Liang et al., 2017; Liang, 2020).

In summary, the functions of rhizosphere and endophytic microbial communities are mainly regulated by plant adaptability. In particular, the functions of rhizosphere microbial communities are mainly affected by MAT, while those of endophytic microbial communities are mainly affected by MAP. The increase in MAT can indeed enhance the activity of rhizosphere microorganisms, which means that the role of rhizo-microorganisms will become ever greater in the context of global warming. The decrease in MAP will enhance the entombing effect of microorganisms, thereby enhancing the carbon sequestration of roots. Overall, global changes will promote the underground carbon sequestration of the root-microbial system in desert steppes, with minimal impact on typical steppes. Meanwhile, it is likely to promote a negative effect in the meadow steppes of cold regions. Therefore, the meadow steppes in cold regions should be the focus of future studies.

## 5 Conclusion

In the present study, the authors demonstrate that the composition and function of the rhizosphere microbial communities were mainly influenced by MAT, while those of the endophytic microbial communities were mainly influenced by MAP. Both rhizosphere and endophytic microbial communities can be divided into two unrelated systems based on their ecological niches. MAT mainly affected the growth, reproduction, and lipid decomposition of rhizosphere microorganisms, whereas MAP mainly affected reverse transcription and cell wall/membrane/envelope biogenic functions of endophytic microorganisms, in the Inner Mongolian steppe ecosystems. The structure and function of rhizosphere and endophytic microbial communities were mainly regulated by plant adaptability.

We recommend that, when studying and addressing the impact of global changes on the root-microbial system, a few populations that are closely related to the root system, such as Rhizobiales, should be paid greater attention in light of their role in carbon sequestration and plant adaptability regulation. Compared to the desert steppe and typical steppe, the root-microbial system in cold regions of the meadow steppe is more vulnerable to the adverse effects of global change, requiring greater measures to address the loss of underground carbon caused by global changes in climate. For example, in case global warming is not curbed, soil moisture content can be reduced by improving drainage efficiency to promote the entombing effect and enhance the carbon sequestration capacity of the root system. The present study provides first-hand data on the composition and function of root and rhizosphere microbial communities in Inner Mongolian steppe ecosystems, making a significant contribution for biogeography and ecology research in the context of global climate change.

## Data availability statement

The datasets presented in this study can be found in online repositories. The names of the repository/repositories and accession number(s) can be found below: https://www.ncbi.nlm.nih.gov/, PRJNA1042678.

## Author contributions

WS: Conceptualization, Data curation, Funding acquisition, Investigation, Methodology, Software, Visualization, Writing – original draft, Writing – review & editing. YW: Investigation, Writing – original draft. BP: Investigation, Writing – original draft. LY: Investigation, Writing – original draft. JG: Funding acquisition, Project administration, Validation, Writing – review & editing. CX: Writing – review & editing.
