# Supplementary material for: Structure and function of microbiomes in the rhizosphere and endosphere response to temperature and precipitation variation in Inner Mongolia steppes
Source: Front Plant Sci. 2023 Dec 7;14:1297399. doi: 10.3389/fpls.2023.1297399 (PMC10733484; doi:10.3389/fpls.2023.1297399)
Supplement: Supplementary file 3 [file Table_3.docx]

Supplementary Material

# SUPPLEMENTARY INFORMATION

Supplementary Table S1. Descriptions of sampling sites.

Supplementary Table S2. eggNOG ID, description, class, and category information of the functional gene database.
